# The Impact of Death Feigning Behaviour on the Survival of Grass Snake (*Natrix natrix*): A Study with Plasticine Models

**DOI:** 10.3390/biology15161432

**Published:** 2026-08-19

**Authors:** Zsuzsanna Gedai, Dávid Szép, Jenő J. Purger

**Affiliations:** 1 Department of Ecology, Faculty of Sciences, University of Pécs, 7624 Pécs, Hungary; gedaizsu@gmail.com (Z.G.); szep.david@pte.hu (D.S.); 2 Department of Ethology, Faculty of Sciences, Eötvös Loránd University, 1117 Budapest, Hungary; 3 Institute of Physiology, Medical School, University of Pécs, 7624 Pécs, Hungary; 4 BioRes Limited Partnership, 7624 Pécs, Hungary

**Keywords:** daily survival rate, predation, prey, thanatosis, wetland

## Abstract

Death feigning (thanatosis) is the antipredator behaviour of grass snake (*Natrix natrix*) which can contribute to its survival. Since this is difficult to study under natural conditions, in our experiment we used four types of plasticine models of grass snake to mimic its different behaviour types. The advantage of using plasticine models is that they preserve the traces of the predators. The study was conducted at two different locations in Kis-Balaton, one of the largest wetlands in the Carpathian Basin. Only 8.75% (*n* = 21) of all grass snake models (*n* = 240) were damaged by predators. The predation activity of birds (76%) was significantly higher than that of mammals (24%), the latter left imprints only on models imitating live (uncoiled, coiled) grass snakes. Among predated models, 62% showed injuries on the midbody, 24% on the tail tip, and 14% on the head. Predation rates were similar in spring and autumn periods, and on the abandoned railway and road. Comparisons of daily survival rates between different model types suggested that the coiled (basking and death feigning) pose may provide an advantage in survival. In some cases, the visible colour pattern of the models may have affected their detectability. The higher survival rates of the grass snake models imitating death feigning indicate the adaptive value of this antipredator behaviour.

## Simple Summary

Our study deals with death feigning (thanatosis) of grass snake to better understand how this antipredator behaviour defense aids survival. Because predation events are difficult to observe directly in the wild, we used four types of plasticine models mimicking different postures of black coloured young grass snake. Overall predator attacks remained low during the study. Birds caused 76% of predation events, while mammals caused 24%. Models mimicking death-feigning or basking poses achieved higher daily survival rates compared to those mimicking grass snake in motion. The results of this study suggest that death-feigning may be an effective, adaptive defense strategy under certain conditions that increases a grass snake’s chances of survival. These findings provide crucial insight into reptile survival mechanisms, which helps improve wildlife conservation strategies and protect biodiversity in fragile wetland ecosystems.

## 1. Introduction

The predator’s goal is to capture and kill its prey, while potential prey has developed a variety of defence mechanisms to survive [1,2]. The variety of defences shows complex interactions and influences the probability of predation [3,4]. Snakes (Serpentes) are predators, but juveniles or small bodied species are often preyed upon by birds or mammals [5,6,7,8,9]. Because of that different types of defence against predators have evolved in most species [1,10]. One such defence is death feigning behaviour also known as thanatosis, when the individual does not show a visible reaction to a stimulus triggered by a predator [11,12,13,14,15]. This defence mechanism can be effective if the killing of prey is not immediately followed by its consumption [14,16]. A sudden change in the prey’s behaviour can stop the predator’s attack because the signal that triggered the attack is missing [17]. In such cases, the predator may become confused and even leave the prey alone for a few moments, which can take advantage of this to escape [18,19]. Field studies have shown that more than half of the grass snake (*Natrix natrix*) individuals exhibited some form of death feigning (the snake curls up, lies on its back, and opens its mouth, often with its tongue protruding and releasing smelly secretion) [20,21,22].

Predation data are difficult to collect in the wild, as these repeated events usually do not overlap in space or time. The predator searches for or waits in ambush for potential prey to appear. The moment of hunting, its success, can therefore only rarely be captured, even with cameras, as we do not know where and when the event occurs [23]. However, plasticine models, such as snakes made of plasticine [7,9,24,25,26,27,28,29], can be used to record predation events, as plasticine preserves the beak and tooth imprints of predators [30]. The method is primarily suitable for detecting diurnal predators (e.g., birds, mammals) that rely on visual cues. The advantage is that a practically unlimited number of models can be used at any chosen location and time [8,31,32]. The identification of predators can be improved by using cameras, but due to the high costs, this method is not cost-effective [33]. The disadvantage is that plasticine models can melt under strong sunlight or high temperatures, they have no body temperature, lack the smell characteristic of natural prey, and they do not move [32,34]. Bird predators also detect their prey based on its movement, so it is more difficult for them to spot the plasticine models due to of their immobility [26].

The effectiveness of death feigning against predators in natural as well as artificial environments is less known [20], but we hypothesized that the efficiency of this antipredator behaviour can be successfully tested using plasticine grass snake models. We presume that the immobility of the death feigning grass snake models should not be a disadvantage, as they imitate a dead individual. Similarly, the plasticine model imitating the moving grass snake used for comparison does not move either, meaning predators can only identify prey based on their shape and colouration.

We performed our study by using plasticine grass snake models with different body postures in a typical grass snake habitat, where the number of potential predator species and their abundance are particularly high, in order to find out: (1) to what extent they are subject to predation and what the role of birds and mammals can be, (2) whether the daily survival rates of dead or death feigning grass snake models are higher than those of grass snake models imitating live individuals that move or bask?

## 2. Materials and Methods

### 2.1. Study Area and Sampling Sites

**Figure 1:**
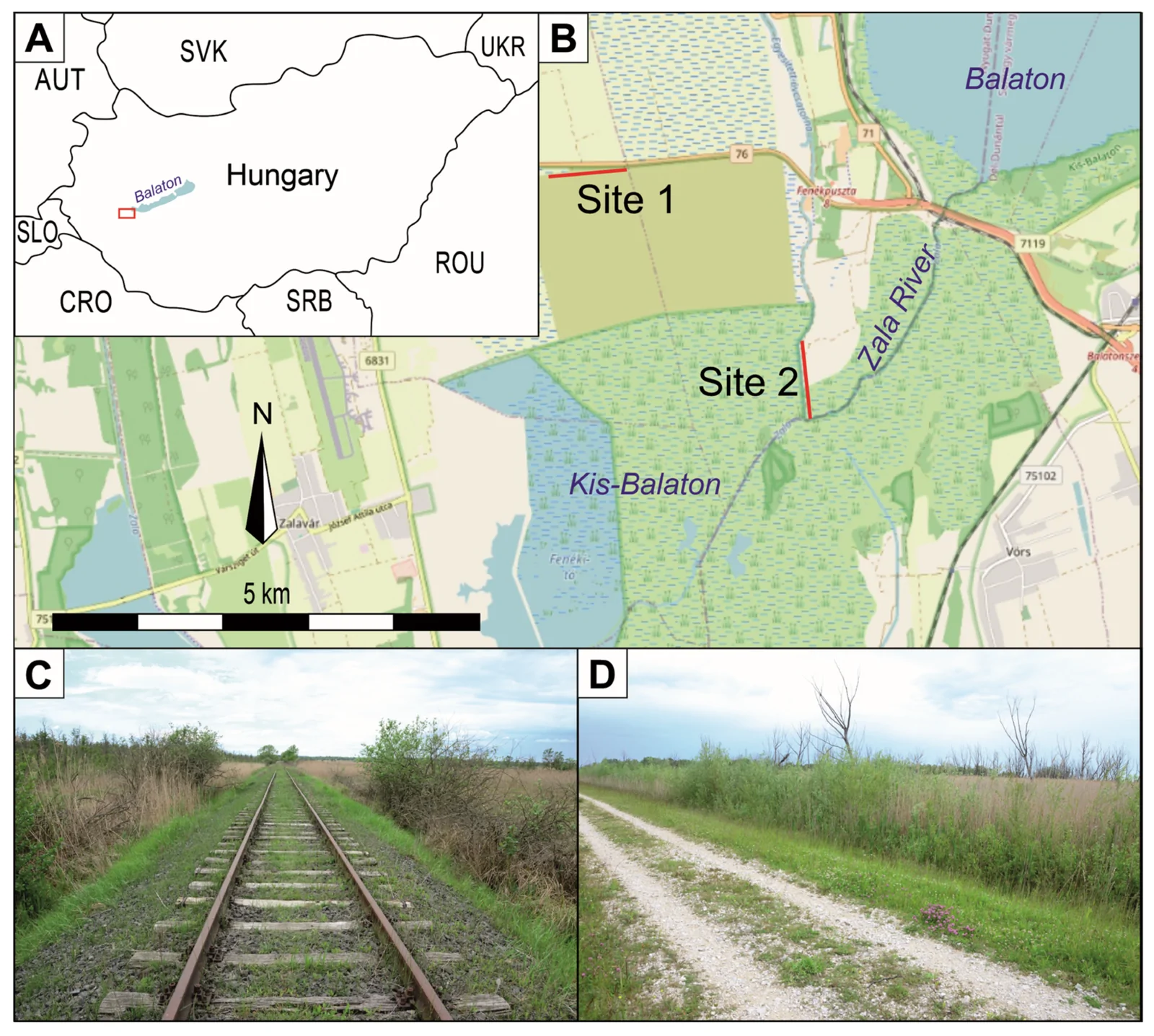
Location of Kis-Balaton (area marked in red rectangle) in Hungary (**A**), then within it at the two study sites the transects are marked with red lines (**B**), views of study sites (**C**,**D**).

The studies were conducted in one of the largest wetlands of the Carpathian Basin, Kis-Balaton, covering an area of approximately 150 km^2^, which has been protected since 1951, and has been a Ramsar site and Important Bird Area since 1979 [35]. A significant part of the wetland area has belonged to the Balaton Uplands National Park since 1997 and has been part of the Natura 2000 (HUBF30003) ecological network (SPA and PSCI) since 2004 [36,37,38]. Furthermore, the important criterion for choosing this area is that the grass snake is one of the most common reptile species in the wetlands of Kis-Balaton [39]. Our study was performed on two embankments approximately 4 km apart (Figure 1). At the first study site, there was an abandoned railway on the embankment (46°42′24″ N 17°11′57″ E), and the second study site was on an embankment with a rarely used gravel road (46°41′08″ N, 17°14′12″ E).

### 2.2. Plasticine Models

**Figure 2:**
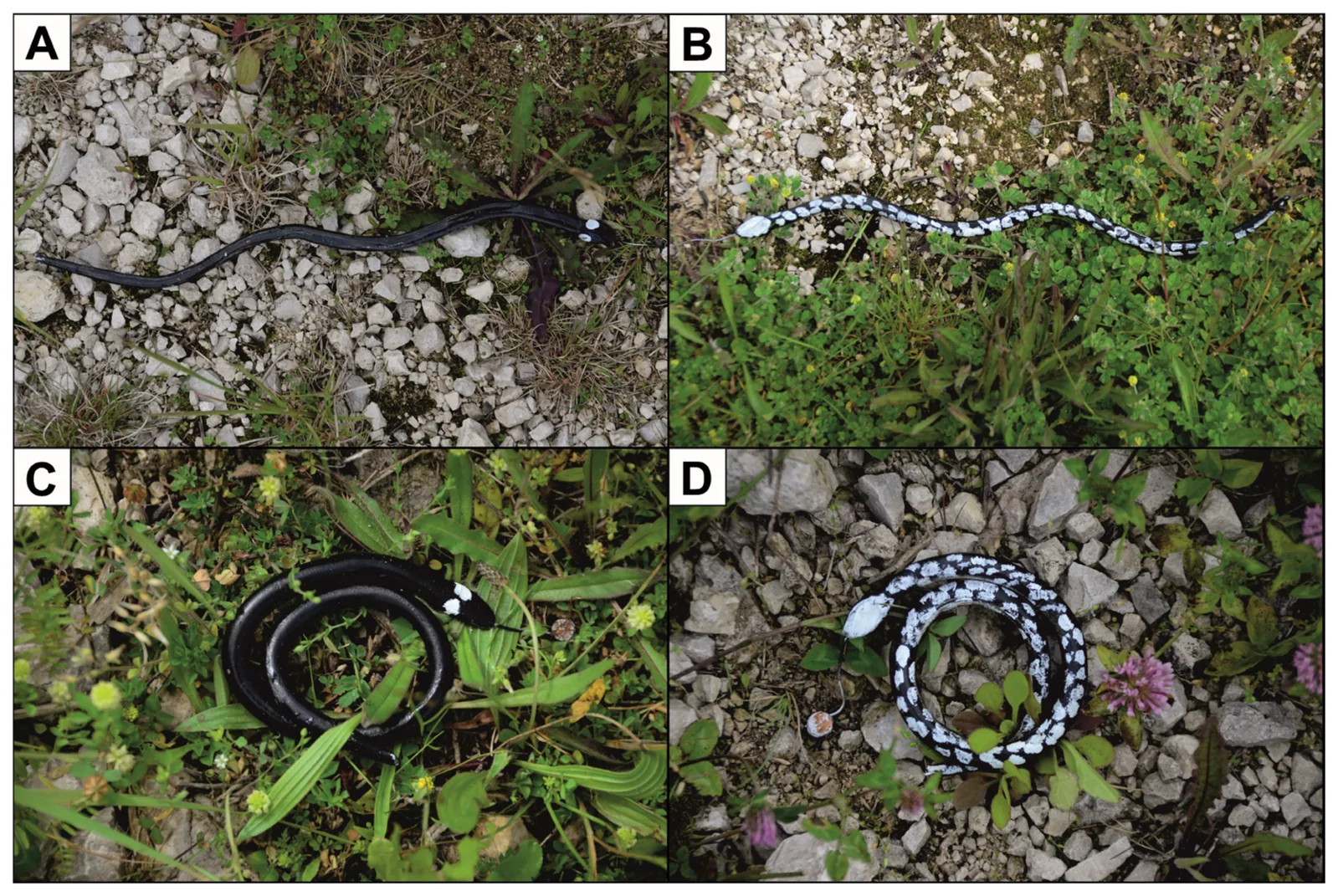
Four types of grass snake plasticine models: (**A**)—uncoiled (live), (**B**)—uncoiled (dead), (**C**)—coiled (basking), (**D**)—coiled (death feigning).

Our first step was making plasticine models to resemble juvenile grass snakes, with a strong contrast between the white collar and the basic body colour (in our case black) [40]. Their venter has a black and white (or yellow) pattern similar to a piano keyboard or a chessboard [39]. We used models imitating young black individuals because they may also attract the attention of smaller potential avian predators (e.g., goshawks, starlings). To give the plasticine model’s support and to be able to fix them, the bodies of the 32 cm grass snake were made on a 40 cm 0.85 mm diameter aluminum wire frame. The 10 mm wide body was gradually tapered towards the tail, the head was widened and slightly flattened. To make one grass snake model, we used approx. 27 g of natural plasticine (KOH-I-NOOR Hardtmuth, České Budějovice, Czech Republic). Of the eight centimetres of wire that was hanging over the head, two to three centimetres were wrapped around a 10 cm nail, which was then driven into the ground or between the pebbles to secure the plasticine model. Each grass snake model was sprayed with a black Plasti Dip rubber layer (Plasti Dip, Blaine, MN, USA), to make the models more resistant to weathering and to mask the unnatural smell of the plasticine [41]. The collar spots and spots on the ventral side were painted white with Plasti Dip. A total of 240 grass snake models were made in four different poses (60 of each, Figure 2): One model type imitated uncoiled live snake (sinusoidal shape) lying on its belly so that only the black grass snake body and the white collar were visible from above and from the side [42]. The second model type was the same in shape as the previous one, but they were lying on their backs, so that from above the grass snake’s belly was visible as a side with light spots, like a dead specimen. The third model type imitated a basking live specimen lying on its belly coiled up [40]. The fourth model type resembles death feigning grass snake, coiled up on its dorsal side, exposing its brightly patterned abdomen (Figure 2). This position is only assumed by grass snakes when a predator is trying to kill them, so it is rarely observed in nature, but it affects the individual’s noticeability.

### 2.3. Study Design

The study began with the placement of the plasticine models on 22 May 2019, and this period overlaps with the breeding season of most bird species in the area. Field observations indicate that grass snakes reach their peak activity levels by late May or early June, driven by the final phases of the mating season and active foraging [43]. The second study period started on 22 September and coincided with the autumn bird migration. From September onwards, the local population density of grass snake noticeable increase due to the synchronized emergence of newly hatched juveniles [44]. We placed 60 models on the abandoned railway and 60 on the embankment in both spring and autumn study periods. We tried to place the models in a location (on railway sleepers, on a gravel road) where the growth of vegetation in spring would have minimal impact on their visibility. At each location, 15 of all four model types were placed randomly at a distance about 10 metres from each other. Consequently, models of the same type were rarely positioned adjacent to one another, thereby reducing the chance that a single predator would attack multiple models. Both studies lasted 15 days and we checked the models 6 times for signs of predation. While placing and inspecting the plasticine models, we wore sterile nitrile gloves to avoid leaving any scent traces. When we detected a sign of predation, we recorded the type and serial number of the model, as well as the date, and took photographs for documentation. The models were left in the field until the end of the experiment, but only the first predation event was considered. Any event, beak, or tooth imprints caused by real predators and left on the plastic model were considered a predation event [45]. Tooth imprints of small rodents [40] and hoof imprints of game animals were not considered. On the last day of the experiments, we collected all plasticine models, and the nails used to attach them.

### 2.4. Data Analysis

A chi-square test was used to compare the predation events between different plasticine models caused by avian and mammalian predators [46]. To account for varying exposure periods, the daily survival rates (DSR) of the models were estimated using Mayfield’s method, which incorporates the exact number of days each model survived [47]. To compare the daily survival rates, the test proposed by Johnson (1979) was applied, and the results were evaluated according to his recommendations [48]. Statistical significance was assessed at the 0.05 level (*p* < 0.05), and all tests were two-tailed.

## 3. Results

### 3.1. Impact of Predators

**Figure 3:**
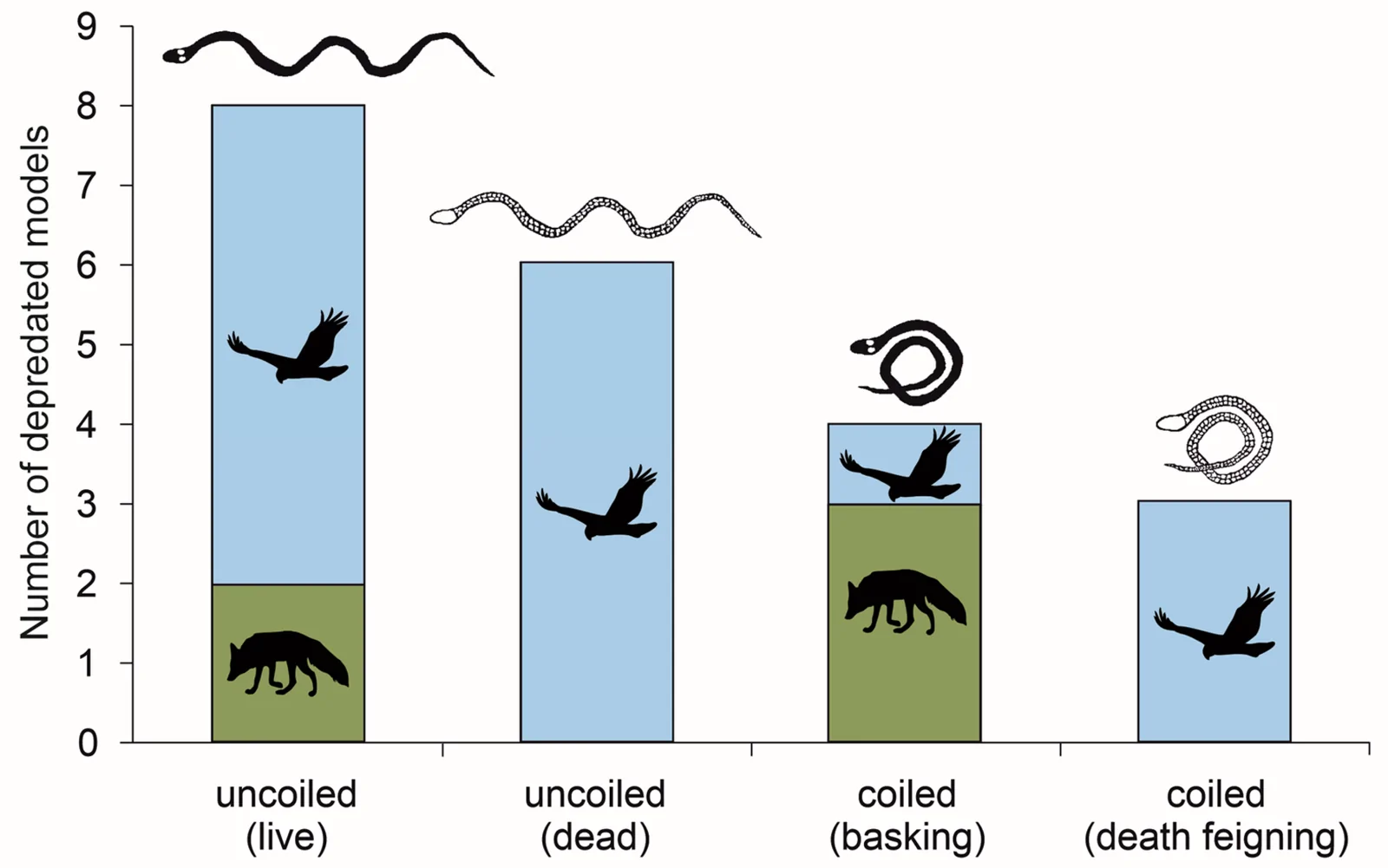
Proportion of bird and mammal predators of different grass snake models.

During the spring and autumn study at the two sites, only 8.75% (*n* = 21) of the plasticine grass snake models (*n* = 240) were damaged by predators. There was no significant difference in the number of predation events among the four different model types (Yates’ chi-square = 1.667; df = 3; *p* = 0.644). The models imitating a live, sinusoidal moving snake lying on their belly, were attacked the most (*n* = 8), followed by the models imitating a sinusoidal shape of dead snake lying on their backs (*n* = 6) (Figure 3).

Models imitating a basking grass snake, coiled lying on their belly (*n* = 4), were attacked less often than models imitating a snake lying on its back, feigning death (*n* = 3) (Figure 3).

Considering both spring and autumn studies, the grass snake models imitating a sinusoidal shape, lying on their belly and backs (*n* = 14), were more than twice as often damaged by predators than the coiled models lying on their backs and belly (*n* = 7), but the difference was not significant (chi-square = 2.333; df = 1; *p* = 0.127).

**Figure 4:**
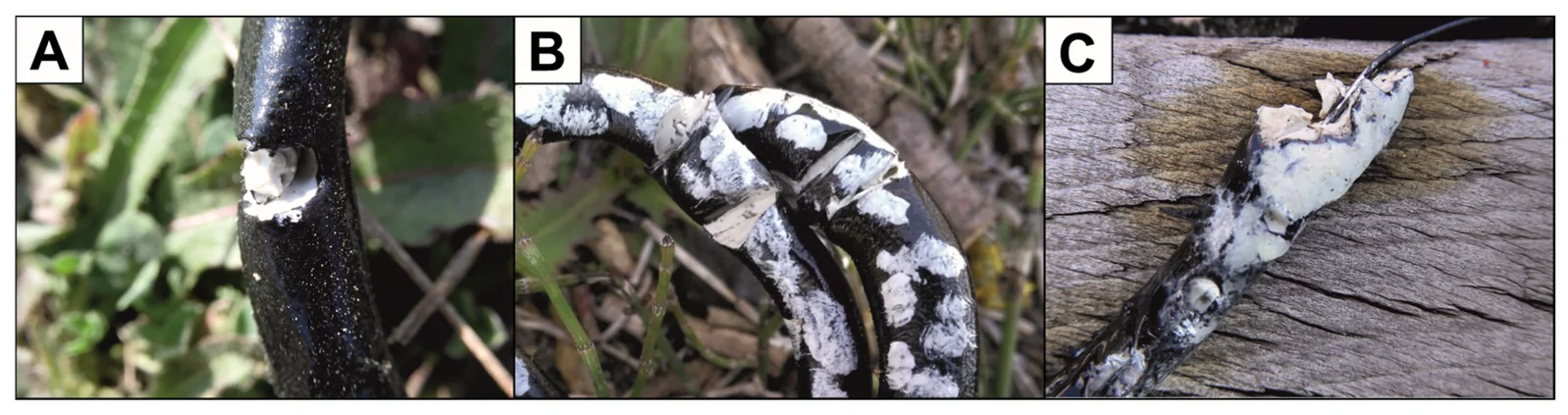
Imprints preserved on the grass snake models: beak of small bird (**A**), beak of larger bird (**B**), and tooth imprints of a mammalian predator (**C**).

Based on the traces left on the plasticine models, the number of predations caused by birds (*n* = 16) was significantly higher (chi-square = 5.762; df = 1; *p* = 0.016) than the number of predations by mammals (*n* = 5). Birds preyed on all model types, but mainly on those imitating a sinusoidal movement (Figure 3), while mammals preyed exclusively on the models imitating living grass snakes lying on their belly (Figure 3 and Figure 4). Of the total predated plasticine models, 62% had injuries primarily on the middle part of the body (*n* = 13), 24% only on the tail tip (*n* = 5), and 14% only on the head (*n* = 3) (Figure 4).

### 3.2. Daily Survival Rates of Different Plasticine Models

Predation rates were similar in spring (*n* = 12) and autumn (*n* = 9) periods, and on the abandoned railway (*n* = 12) and road (*n* = 9) (chi-square = 0.429; df = 1; *p* = 0.513).

Regardless of whether the models were lying on their backs or their belly, the predation rates of uncoiled models (*n* = 14) and coiled models (*n* = 7) did not show significant differences (chi-square = 2.333; df = 1; *p* = 0.127). However, comparisons of daily survival rates between different model types suggested that in some cases the coiled pose may provide an advantage in survival. The daily survival rate (DSR = 0.999; SE = ±0.001) of the coiled models (*n* = 1) placed on the gravel road (basking, death feigning) was significantly higher (Z = 2.387; *p* = 0.017) than the models imitating sinusoidal movement (uncoiled, *n* = 8) or shape (live and dead) (DSR = 0.991; SE = ±0.003). In contrast, on the abandoned railway, coiled (*n* = 6) and uncoiled (*n* = 6) models were damaged by predators in equal proportions.

In some cases, the colour of the models may have affected their detectability. Comparing the daily survival rates of the four grass snake models, significant correlations were detected only during the autumn study on the abandoned railway in two cases: the models imitating a dead snake (uncoiled, lying on the back) (DSR = 1) and the death feigning models (coiled, lying on the back) (DSR = 1) were not predated at all (*n* = 0), so their 100% daily survival rates were significantly higher (Z = 2.02; *p* = 0.043) than those imitating a moving live grass snake (*n* = 4) (DSR = 0.980; SE = ±0.010).

## 4. Discussion

### 4.1. Birds and Mammals as Predator of Grass Snakes

Our results suggest that plasticine models imitating a sinusoidal movement were attacked by predators more often than models with a coiled body shape, regardless of whether they imitated basking or death feigning grass snake. Despite the low predation rates, our results showed that birds as predators were a greater threat to grass snakes than mammals. The beak imprints were found on all types of plasticine models, though birds preferred the uncoiled models with a sinusoidal shape. Potential avian predators of diurnal grass snake primarily respond to visual stimuli when searching for prey [49,50]. Several known natural avian predator of grass snake [40,51,52,53] breed in the study area, such as common pheasant (*Phasianus colchicus*), great crested grebe (*Podiceps cristatus*), Eurasian bittern (*Botaurus stellaris*), black-crowned night heron (*Nycticorax nycticorax*), cattle egret (*Bubulcus ibis*), grey heron (*Ardea cinerea*), purple heron (*Ardea purpurea*), white stork (*Ciconia ciconia*), barn owl (*Tyto alba*), little owl (*Athene noctua*), common buzzard (*Buteo buteo*), European honey buzzard (*Pernis apivorus*), black kite (*Milvus migrans*), western marsh harrier (*Circus aeruginosus*), northern raven (*Corvus corax*), hooded crow (*Corvus cornix*), Eurasian magpie (*Pica pica*), Eurasian jay (*Garrulus glandarius*), common starling (*Sturnus vulgaris*), common blackbird (*Turdus merula*). Based on the beak imprints preserved on plasticine grass snake models, it is not possible to identify the birds to the species level, and at most, we can only deduce the body size of the bird. During our study, we mostly saw flocks of starlings on the embankments, and in most cases, the size and shape of the imprints preserved on the plasticine models resembled the common starling’s beak imprint.

In our study it was found that mammals only preyed on the uncoiled models, from which it can be assumed that they associated the sinusoidal appearance of the models with that of moving grass snakes, and this is certainly the stimulus that could have triggered predation [17]. The lack of predation events caused by mammals in this study can be explained by the fact that the plasticine models were scentless and immobile, while most predatory mammals use olfactory cues in their hunting during the night, in addition to visual stimuli of movement during the day [32,54,55]. The use of camera traps could improve the identification accuracy of potential predators and account for events where they detect the model without harming it [33]. The tooth marks left in the plasticine were not suitable for identification of predators at the species level, because the small plasticine grass snakes preserved only fragments of the tooth marks of larger mammals, or only the chewed parts of the model remained. Among the mammals in the study area, the omnivorous wild boar (*Sus scrofa*) and the red fox (*Vulpes vulpes*) consume snakes [36,56], and remains of grass snakes occasionally are found in the diet of the golden jackal (*Canis aureus*), the otter (*Lutra lutra*) and the domestic cat (*Felis catus*) [36,57,58,59]. The study area lacks venomous snakes that would elicit predator avoidance behaviours [39].

### 4.2. Shapes and Colours of Grass Snake Models Influence Their Detectability

Uncoiled models were attacked more often than coiled models, but significant correlations were found only in one case when comparing the daily survival rates of each model type. Specifically, on the gravel road, the daily survival rate of coiled models (representing basking and death-feigning behaviors) was significantly higher than that of models imitating sinusoidal movement or shape (representing live and dead snakes). Grass snakes are known to bask in a coiled position [20,60], which is not only important for thermoregulation [61], but may also be beneficial in reducing the chance of predation. Grass snakes with a coiled body can blend in better with the environment and, since they do not move, predators have difficulty identifying them as potential prey, and this is even more true for smaller or young snakes [8]. This may explain why the coiled grass snake models were either not found or did not appear to be prey.

We found that in autumn, on the abandoned railway, the daily survival rate of models imitating dead (sinusoidal shape, lying on the back) and dead feigned (lying on the back) grass snake was significantly higher than that of models imitating live moving grass snake lying on the belly. The later models with uniform blackness, which is interrupted only by the white nape patch, probably could be more easily noticed on the plain grey of the abandoned railway, by both avian and mammalian predators. Sometimes even very small colour differences can influence the outcome of predation, e.g., [40] showed that completely black grass snake models were significantly more attacked than “normal” models with a white collar on a black background. Regardless of their shape, the spotted venter of a grass snake model lying on its back breaks the outline of the individual and makes it more difficult to see the entire animal. This phenomenon is even more effective when the animal’s background contains different colours and shapes [2]. The significant result may also be attributed to the increased visibility of young grass snakes in autumn, as they hatch at the end of summer [44].

Based on our results, we suggested that the coiled shape and the black-and-white pattern (typical for the belly which can be seen on dead individual) provide the grass snake with a higher chance of survival, thus death feigning pose may be an effective defence strategy. Plasticine grass snake models cannot reproduce all the elements of death feigning, such as the gaping mouth, the limp body and foul-smelling discharge [20,62], but these more subtle movements may be important when the grass snake imitates death in the presence of a predator. If a live snake that is death feigning is carried to its nest or den by a bird or mammal predator, it may also have a chance to escape quickly before the predator’s offspring eat it. It is not clear whether the predator can recognize a live (simple defending), a death feigned, or a truly dead individual. Some predators are reluctant to attack or continue to attack if their prey is immobile because movement as a visual stimulus is eliminated [63]. Immobility may be effective against predators that rely on visual cues to locate their prey, such as most birds of prey [16]. A sudden change in prey behaviour can be surprising, for example, snakes become immobile when they cannot escape the predator or other defence mechanisms are not effective [11,64]. Plasticine models do not move, so the predator must decide whether to attack or not during detection. Tonic immobility is often used as a synonym for death feigning [18,65,66,67], but the latter is much more complex [20,68,69]. Both behaviours are considered fear-mediated responses [70,71]. The question has arisen whether this frequently observed immobility serves a function of death feigning. Several animal species exhibit the mechanism of immobility, but in this state, they do not resemble a dead individual, perhaps because that is not their purpose, and therefore these two mechanisms should be treated completely separately [20,66].

Essential information for understanding evolutionary processes, such as adaptation, can be obtained in a study with plasticine grass snake models since it is very difficult to directly study predation events in nature. This method can only be used for the identification of predators and to estimate the predation pressure but is not suitable for showing whether the predator would consume the prey or whether the potential prey would manage to escape.

## 5. Conclusions

Our study demonstrates that while clay models of grass snake primarily help detect visually hunting predators, they provide insights into the survival value of specific body postures and coloration. Although predation rates on stationary clay models are inherently lower than on live, moving prey, our findings confirmed that the death feigning posture offers a distinct survival advantage under certain conditions. However, because static models cannot replicate the full complexity of thanatosis, these results should not be used to evaluate the overall effectiveness of dynamic death feigning behavior; instead, they serve specifically to isolate and evaluate predator responses to distinct prey body postures.

## Data Availability

Data are contained within the article.
